# Supplementary material for: Learning curve and surgical outcome of robotic assisted colorectal surgery with ERAS program
Source: Sci Rep. 2022 Nov 29;12:20566. doi: 10.1038/s41598-022-24665-w (PMC9709162; doi:10.1038/s41598-022-24665-w)
Supplement: Supplementary file 2 — Supplementary Information 2. [file 41598_2022_24665_MOESM2_ESM.zip › RAL Raw data-Ñ[▒K/047/HS_ñw╝╨░O▒Kñσ.pdf]

正式摘要(電子簽章：000000206983678)

W52 / W52 ☐ 英文標題(注意:無英文姓名-請補病人基本檔!) ☐【讀取】憑證

臺中榮民總醫院

出院病歷摘要

轉入號 : 轉出號 :

[redacted]

(8)流水編號	
(9)入院日期	109/12/22 (2020) 直腸外科 W52 - 121
(10)轉科日期	
(11)出院日期	109/12/31 (2020) 住院天數計 9 日

診斷

- 入院 : 1. Rectum tumor, FAV 5cm, cT3 N1 M0, Stage IIIB,
  - s/p 5-FU(800/m2) on 2020/10/06 11/02, 11/22
  - s/p Tomo IMRT *5 on 2020/10/5-13
  - s/p RA-3 IMRT*25 on 10/15-11/23
  2. HTN, under Norvasc and Cardolol
  3. BPH, under Harnalidge
  4. Duodenum ulcer
- 出院 : 1. Rectum tumor, FAV 5cm, cT3 N1 M0, Stage IIIB,
  - s/p 5-FU(800/m2) on 2020/10/06 11/02, 11/22
  - s/p Tomo IMRT *5 on 2020/10/5-13
  - s/p RA-3 IMRT*25 on 10/15-11/23
  - s/p Robotic LAR + loop ileostomy on 2020/12/23
  - ypT2N0(0/16)M0, stage I,
  - MD, AngI-, PI-, TuDp-, CRM: 3cm-, PrM: 9.7cm, DisM: 7mm, ExN-, TRG:2,
  2. HTN, under Norvasc and Cardolol
  3. BPH, under Harnalidge
  4. Duodenum ulcer

主訴

- constipation and blood tinged stool since 2020/08

病史

- The 63 years old male had medical history of
  1. Rectum tumor, FAV 5cm, cT3 N1 M0, Stage IIIB,
  - s/p 5-FU(800/m2) on 2020/10/06 11/02, 11/22
  - s/p Tomo IMRT *5 on 2020/10/5-13
  - s/p RA-3 IMRT*25 on 10/15-11/23
  2. HTN, under Norvasc and Cardolol
  3. BPH, under Harnalidge
  4. Duodenum ulcer
  Initially, he suffered from constipation and blood tinged stool since 2020/08. Thus, he visited VGH PuLi in 2020/8, where he received COS to cecum on 2020/09. A rectum tumor, 5 cm FAV was noted by the COS. Biopsy was done, the pathology showed Tis, adenocarcinoma. Under such condition, he was referred to our hospital for further evaluation.
  On 2020/09/29, we rechecked sigmoid scope. The rectum tumor was noted at 5cm FAV. Biopsy showed adenocarcinoma, moderately differentiated. He received pelvis MRI for further staging. No distant meta was noted. Further survey such as Liver MRI and PET whole body scan was arranged.
  Under the impression of Rectum tumor, FAV 5cm, cT3 N1 M0, Stage IIIB, he received neoadjuvent CCRT since 2020/10/05. The 3rd CCRT was done on last admission 2020/11/23. This time, he was admited for scheduled surgery of robotic assisted LAR.

體檢發現

- CEA 1.52(2020/9)

  2020/9 COS to cecum, s/p Px *3. rectum tumor 5 cm FAV
  2020/8 lung abd CT: rectum tumor. GBS

- 2020/9 pelvis MRI: Lt obturator LN 283.
  2020/10 liver MRI: Neg. GBS
  patho: Tis, adenocarcinoma 9/30 adenocarcinoma
  - General appearance :
    - easy looking , conscious clear , without cardiopulmonary distress
    - Vital signs: B.P. 127/76 mmHg , P.R. 88 /min , R.R. 17 /min , B.T. 36.7 ℃
  - HEENT :
    - Eyes: no anemic conjunctiva, no icteric sclera, normal EOM
    - ENT: no otorrhea, no rhinorrhea, no erythematous mucosa, no injected tonsil, no deformity
  - Neck: supple, freely movable, trachea midline,no deformity, no engorged jugular vein , no palpable thyroid or lymph node enlargement
  - Chest:
    - Inspection: Grossly normal, symmetric and fully expansion, no accessory muscle used, no operation scar, no spider angiomata, no gynecomastia
    - Palpation: no palpable mass or nodule, no subcutaneous emphysema, no tenderness
    - Ausculation: clear breathing sound
  - Heart:
    - Percussion: normal heart size
    - Ausculation: regular heart beat, normal S1, S2, no gallop, no murmur
  - Abdomen:
    - Inspection: in
      - flat contour, symetrically, no scar, no stria, no dilated veins, no rashes
    - Ausculation: normoactive bowel sound, no abdominal bruits
    - palpation: soft, no muscle guarding or rigidity, no local tenderness, no rebounding pain, no palpable mass, no palpable pulsation
    - Percussion: no shifting dulllness, no hepatomegaly, no splenomagaly
  -

Peripheral pulsation :	CA	BA	RA	FA	PA	PTA	DPA
Right side :	++	++	++	++	++	++	++/+++
Left side :	++	++	++	++	++	++	++/+++

  - Genito-anus: no tenderness or palpable mass lesion or rectal shelf, normal external genitalia, normal anal tone, brown stool

CRS CONDITION

  -
    - Lung

手術日期及方法

- 手術日期及方法20201223 Robot Assisted Low Anterior Resection + loop ileostomy

住院治療經過

- After admission, Robotic LAR + loop ileostomy was performed on 2020/12/23. He recovery well and can tolerance bland II diet . He was discharged on 12/31.

合併症

一般檢查

- 累積報告 :

DATE	TIME	ALB	TP	BIL.T	BIL.D	ALKP	AST	ALT	LDH	CHOL	TG	HDL-C	LDL	UA	NH3	AMY
1091222	1601			0.91			22	19								
1091223	1626			1.10	0.24		20	16								
1091228	1024			0.48			14	7								

DATE	TIME	WBC	RBC	HGB	HCT	MCV	PLT	BLST	NEUT	BAND	SEG	LYM	MON	EOS	BAS
1091222	1602	3930	4.32	13.8	38.9	90.0	195	0.0		0.0	55.0	22.0	14.0	7.0	2.0
1091223	1624	7300	4.27	12.9	38.2	89.5	174		90.1			6.3	3.3	0.0	0.3
1091228	1026	4690	3.60	11.6	32.7	90.8	162		69.3			11.9	11.5	6.4	0.9

DATE	TIME	NA	K	CL	CA	BUN	CREAT	Mg	P	CRP	ESR	PCT	LIPASE	LACTATE
1091222	1601	142	4.4	107	9.1	11	1.04							
1091223	1626	141	4.3	109	8.6	13	1.09							
1091228	1024	140	3.7	108	8.1	14	0.83							

DATE	TIME	GLUCOSE	PAB	TRF	ZN	CU	KETONE	CK	CKMB	PT-P	PT-C	APTT-P	APTT-C	TRO-T
1091222	1601	91												
1091222	1602									10.2	10.3	26.3		

- 放射線報告 :**檢驗項目 :CHEST AP VIEW (PORTABLE) 報告日期 :1091224**
  主 訴：
  Result:
  Finding:
  > Enlarged cardiac silhouette
  > Calcification of thoracic aorta
  > Mildly increased insterstitial infiltration over bilateral lower lung fields
  > Blunting of bilateral CP angle, may be due to pleural effusion or pleural change
  > Degenerative change of thoracic and lumbar spine with osteophytes formation.
  > On nasogastric tube, chemo port and central line
  Impression:
  As above description.

特殊檢查

病理報告

- 活體組織切片病理報告 手術日期：**109/09/15** 病理號：**S0928841**

診斷醫師:王任卿 報告日期:109/09/21
Pathologic diagnosis:
Rectum, 5 cm from anal verge, endoscopic biopsy --- Adenocarcinoma
in situ arising in tubulovillous adenoma.
Ancillary study for diagnosis:
Recut and IHC stain for AE1/AE3 done.
Prognostic and predictive factor:
1. Degree of dysplasia: High grade.
2. Margin of stalk: Cannot be well evaluated.
Gross description:
The specimen consists of a piece of tan soft tissue, 0.6x0.5x0.3 cm.
All for section.
Microscopic description:
Section shows colon mucosa with tubulovillous glands proliferation
with dysplasia. Complex cribriform glands with abortive structure
seen but not beyond muscularis mucosa.
Note:
1. This case has been peer reviewed by two doctors.
2. The diagnosis is concurred in intradepartment consensus meeting
on 2020/09/21.
#T-59300_2 #M-82630_2 0 1340 00CM00
#T-59300_2 #M-81402_2 9 1340 000000

其它

出院時情況

- 3.改門診治療

出院指示

藥名	劑量	劑量單位	用法	頻次	數量	數量單位	現況	天數	單位
CR Stilnox tab6.25	1	TAB	PO	HS	5	TAB	TH	5	天
OCAS Harnalidge 0.4 mg	1	TAB	PO	QDAC	5	TAB	TH	5	天
Ulstop tab 20mg	1	TAB	PO	BID	10	TAB	TH	5	天
Ultracet tab	1	TAB	PO	Q6HPRN	20	TAB	TH	5	天
Loperamide cap 2mg	1	CAP	PO	BID	10	CAP	TH	5	天

出院計劃

主治醫師 2222H 林俊余 / 住院醫師 9761G 王文謙
正式摘要(電子簽章：**000000206983678)**

W52 / W52

doctorRank:B
